# Association between carotid plaque progression and persistent endothelial dysfunction in an infarct-related coronary artery in STEMI survivors

**DOI:** 10.1007/s00380-024-02444-z

**Published:** 2024-07-27

**Authors:** Takeo Horikoshi, Takamitsu Nakamura, Ryota Yamada, Toru Yoshizaki, Yosuke Watanabe, Manabu Uematsu, Tsuyoshi Kobayashi, Akira Sato

**Affiliations:** Department of Cardiology, Faculty of Medicine, University of Yamanashi, 1110 Shimokato, Chuo, 409-3898 Japan

**Keywords:** Endothelial function, Carotid plaque, Myocardial infarction

## Abstract

Persistent coronary endothelial dysfunction predicts future adverse events; however, performing multiple invasive endothelial function tests is difficult in actual clinical practice. This study examined the association between carotid plaque progression and persistent coronary endothelial dysfunction using serial assessments of the coronary vasomotor response to acetylcholine (ACh) in the infarct-related artery (IRA) among patients with ST-elevation acute myocardial infarction (STEMI). This study included 169 consecutive patients with a first STEMI due to the left anterior descending coronary artery (LAD) occlusion who underwent successful percutaneous coronary intervention. The vasomotor response to ACh in the LAD was measured within two weeks after acute myocardial infarction (AMI) (first test) and repeated at six months (second test) after AMI. Ultrasonography of the bilateral common carotid artery and internal carotid artery was performed during the acute phase, and the thickest intima-media thickness (IMT) of either artery was measured as the maximum IMT. After six months, the IMT at the site of maximal IMT was re-measured to determine the carotid plaque progression. Finally, 87 STEMI patients analyzed. At 6 months, 25 patients (28.7%) showed carotid plaque progression. In a multivariable analysis, carotid plaque progression was identified as an independent predictor of persistent coronary endothelial dysfunction, both in terms of coronary diameter response [odd ratio (OR) 3.22, 95% confidence interval (95% CI) 1.13–9.15, *p* = 0.03] and coronary flow response [OR 2.65, 95% CI 1.01–7.00, *p* = 0.04]. Independently, carotid plaque progression is linked to persistent endothelial dysfunction in the IRA among STEMI survivors.

**Supplementary Information:**

The online version contains supplementary material available at 10.1007/s00380-024-02444-z.

## Introduction

Coronary endothelial function is impaired after myocardial infarction (AMI), with a more intense endothelial dysfunction, particularly in infarct-related coronary arteries (IRA) [1]. This causes endothelial injury, leading to endothelial dysfunction in the entire vascular tree of the IRA [2–5]. We have previously reported that acute coronary endothelial dysfunction after AMI results in an altered vascular endothelial response over time. This phenomenon suggests that in patients with AMI, a single acute assessment of coronary endothelial function does not necessarily reflect the risk of future events. Additionally, coronary endothelial dysfunction persisted during the chronic phase in some patients despite optimal medical therapy (OMT) [4].

Whether one-point coronary endothelial dysfunction is known to be associated with future MACE [6, 7], we have previously reported that persistent coronary endothelial dysfunction in the chronic phase after AMI is an independent predictor of increased plaque in the responsible coronary artery and cardiovascular prognosis [8, 9]. Although carotid plaque is known to be associated with vascular endothelial dysfunction, as measured by brachial flow-mediated dilatation (FMD) [10], there have been no previous studies on persistent endothelial dysfunction and carotid plaque in the directly measured IRA.

It is important to noninvasively and indirectly assess persistent coronary endothelial dysfunction, which is an independent prognostic predictor. Indirectly assessing this condition using simple carotid echocardiography may help identify patients who require more intensive interventions to improve their prognosis. In the present study, we investigated the association between carotid plaque changes and persistent coronary endothelial dysfunction in patients with ST-elevation acute myocardial infarction (STEMI) with the left anterior descending coronary artery (LAD) as the culprit lesion.

## Methods

### Study patients

This study was a sub-analysis of data from a prospective cohort observational study registered at URL: https://upload.umin.ac.jp/cgi-open-bin/ctr/ctr_view.cgi?recptno=R000021343 (unique identifier: UMIN000018434). Initially, it included 169 consecutive patients with a first STEMI due to occlusion of the proximal segment of the LAD, who were admitted to the Yamanashi University Hospital between January 2007 and December 2017. All patients underwent emergency coronary angiography and successful reperfusion therapy within 12 h of symptom onset by primary percutaneous coronary intervention (PCI). The diagnosis of STEMI was based on the presence of each of the following criteria [11]: typical chest pain persisting for ≥ 30 min, ST-segment elevation ≥ 0.2 mV in two or more contiguous leads on a standard 12-lead electrocardiogram (ECG), and creatine kinase-MB level ≥ two-fold of the upper limit of normal or troponin T level > 0.1 ng/mL. The inclusion criteria were the absence of organic residual stenosis > 30% in the LAD for moderate stenosis in the conduit coronary artery, which may cause an increase in the coronary blood flow [12] and patients who underwent carotid echocardiography twice, once in the acute phase, and once in 6 months after AMI. The exclusion criteria were as follows: (1) previous PCI in the LAD; (2) previous coronary artery bypass surgery; (3) organic stenosis ≥ 30% in the LAD at either 1–2 weeks (first test) or 6 months (second test) after AMI; (4) cardiovascular events during the 6-month follow-up period between the first and second tests after AMI; (5) epicardial coronary constriction degree > 30% in the LAD in response to acetylcholine chloride (ACh); (6) presence of collaterals to the LAD with Rentrop grade ≥ 2; 7) congestive heart failure (New York Heart Association classification IV) at one week after AMI; (8) persistent atrial fibrillation and a paced rhythm; (9) age > 80 years; (10) presence of valvular heart diseases (aortic stenosis [aortic valve area < 1.0 cm^2^], aortic regurgitation [Sellers classification III/IV], or mitral regurgitation [Sellers classification III/IV]), secondary hypertension, stroke, renal dysfunction (serum creatinine > 2.0 mg/dL), or other serious diseases. Finally, 87 patients were included in the study. Written informed consent was obtained from all patients prior to the study. This study was approved by the Ethics Committee of the Yamanashi University Hospital and conformed to the principles outlined in the 1975 Declaration of Helsinki.

## Study protocol

**Fig. 1:**
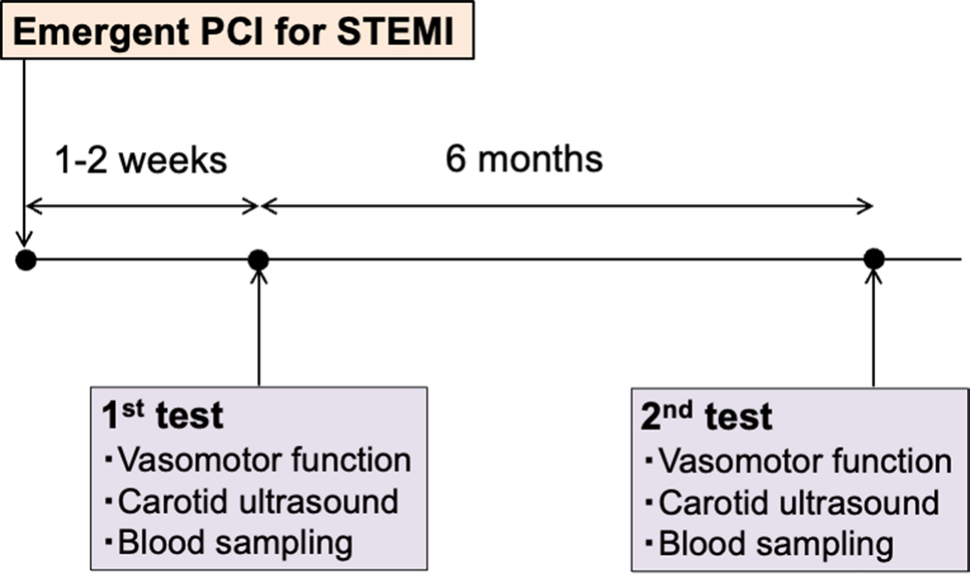
Timeline of clinical examination and follow-up

This study aimed to assess the predictive value of carotid plaque progression for persistent coronary endothelial dysfunction in the IRA. After emergent coronary angiography with PCI at admission, cardiac catheterization, including coronary vasomotor function tests, was repeated at 1–2 weeks and 6 months (first and second tests, respectively) (Fig. 1). The patients received standard medical treatment after admission [13], which was continued throughout the follow-up period, as shown in Table 1. Recommended lifestyle and diet changes were implemented throughout the follow-up period. Vasodilators, including calcium channel blockers, nitrates, and nicorandil, were withdrawn more than 3 days before the coronary vasomotor function test. Blood samples were collected from the peripheral vein early in the morning, a few days before discharge from the hospital, during the first and second tests.

**Table 1:** Comparisons of Clinical Characteristics between Patients with and without Carotid Plaque Progression

	Total (*n* = 87)	Plaque Progression		*p* value
		With (*n* = 25)	Without (*n* = 62)	
Age, years	65 (57, 73)	64 (47, 49)	66 (58, 73)	0.15
Sex, male, no (%)	70 (80.5)	21 (84.0)	49 (79.0)	0.77
Hypertension, no (%)	56 (64.4)	17 (68.0)	39 (62.9)	0.65
Diabetes mellitus, no (%)	21 (24.1)	9 (36.0)	12 (19.4)	0.10
BMI, kg/m^2^	23.5 (22.0, 26.1)	24.3 (20.6, 27.6)	23.3 (22.1, 25.4)	0.28
Current smoking, no (%)	41 (47.1)	12 (48.0)	29 (46.8)	0.92
TIMI risk score 2°P	2 (1, 2)	2 (1, 2)	2 (1, 2)	0.58
Measurements at the 1st test				
Systolic BP, mmHg	122 (109, 140)	125 (101, 141)	120 (110, 140)	0.97
Diastolic BP, mmHg	70 (62, 79)	72 (60, 80)	68 (62, 79)	0.58
HbA1c, %	5.9 (5.7, 6.6)	5.9 (5.5, 6.9)	6.0 (5.7, 6.3)	0.75
Triglyceride, mg/dL	138 (108, 180)	147 (120, 175)	131 (107, 181)	0.39
HDL-C, mg/dL	39 (34, 46)	41 (33, 47)	38 (34, 46)	0.47
LDL-C, mg/dL	134 (113, 161)	140 (116, 164)	129 (113, 160)	0.59
Apo B/A1	0.99 (0.84, 1.23)	0.99 (0.83, 1.22)	0.99 (0.84, 1.25)	0.98
Apo C2, mg/dL	5.0 (4.1, 6.5)	5.5 (4.9, 6.9)	4.7 (3.9, 6.3)	0.04
Apo C3, mg/dL	8.6 (7.3, 10.5)	9.0 (7.6, 11.1)	8.6 (7.2, 10.4)	0.28
Apo E, mg/dL	4.5 (4.1, 5.6)	5.6 (4.2, 6.1)	4.4 (4.0, 5.1)	0.01
RLP-C, mg/dL	4.9 (3.5, 7.3)	5.1 (4.1, 7.5)	4.7 (3.5, 7.3)	0.40
Lp(a), mg/dL	23.1 (12.5, 48.4)	22.0 (12.3, 48.9)	24.6 (11.1, 47.0)	0.80
hsCRP, mg/dL	0.60 (0.19, 1.07)	0.66 (0.33, 1.85)	0.48 (0.18, 1.03)	0.29
BNP, pg/mL	87 (29, 216)	71 (24.8, 112)	123 (30, 246)	0.20
LVEF, %	57 (48, 64)	56 (47, 62)	57 (48, 65)	0.43
Carotid Plaque, mm	1.4 (1.0, 2.2)	1.3 (0.9, 2.3)	1.5 (1.0, 2.0)	0.50
Measurements at the 2nd test				
Systolic BP, mmHg	124 (110, 137)	126 (110, 139)	121 (111, 136)	0.99
Diastolic BP, mmHg	72 (64, 80)	75 (65, 82)	71 (64, 79)	0.42
HbA1c, %	6.0 (5.8, 6.5)	5.9 (5.7, 6.7)	6.1 (5.8, 6.5)	0.44
Triglyceride, mg/dL	133 (92, 189)	148 (91, 236)	125 (92, 179)	0.23
HDL-C, mg/dL	44 (38, 50)	42 (36, 50)	44 (39, 50)	0.69
LDL-C, mg/dL	85 (68, 101)	84 (73, 101)	85 (67, 101)	0.63
Apo B/A1	0.63 (0.50, 0.72)	0.64 (0.53, 0.73)	0.63 (0.48, 0.72)	0.66
Apo C2, mg/dL	4.3 (3.3, 5.3)	4.7 (3.7, 7.0)	4.1 (3.1, 5.1)	0.07
Apo C3, mg/dL	9.3 (7.7, 11.5)	10.5 (8.3, 14.1)	9.0 (7.3, 10.6)	0.04
Apo E, mg/dL	4.0 (3.3, 5.1)	4.9 (3.6, 5.5)	3.8 (3.2, 5.1)	0.09
RLP-C, mg/dL	4.6 (2.9, 6.9)	6.2 (3.8, 8.5)	4.3 (2.8, 6.3)	0.02
Lp(a), mg/dL	14.1 (6.0, 25.9)	13.2 (5.5, 29.4)	15.0 (6.2, 26.3)	0.94
hsCRP, mg/dL	0.06 (0.03, 0.16)	0.06 (0.04, 0.37)	0.07 (0.03, 0.16)	0.50
BNP, pg/mL	29 (20, 66)	27 (10, 146)	31 (21, 62)	0.58
LVEF, %	59 (51, 68)	54 (47, 69)	59 (52, 67)	0.37
Carotid Plaque, mm	1.3 (0.9, 2.0)	1.6 (1.3, 2.7)	1.2 (0.8, 1.8)	< 0.01
Medications at the 1st test, no (%)				
Beta-blocker	37 (42.5)	11 (44.0)	26 (41.9)	0.86
ACE-I/ARB	60 (69.0)	18 (72.0)	42 (67.7)	0.70
Ca blocker	56 (64.4)	15 (60.0)	41 (66.1)	0.59
Statin	80 (92.0)	24 (96.0)	56 (90.3)	0.67
Diuretics	8 (9.2)	3 (12.0)	5 (8.1)	0.68
Insulin	2 (2.3)	1 (4.0)	1 (1.6)	0.50
Medications at the 2nd test, no (%)				
Beta-blocker	39 (44.8)	11 (44.0)	28 (45.2)	0.92
ACE-I/ARB	64 (73.6)	18 (72.0)	46 (74.2)	0.83
Ca blocker	60 (69.0)	16 (64.0)	44 (71.0)	0.53
Statin	83 (95.4)	24 (96.0)	59 (95.2)	1.00
Diuretics	13 (14.9)	4 (16.0)	9 (14.5)	1.00
Insulin	2 (2.3)	1 (4.0)	1 (1.6)	0.50
PCI variables				
Peak CPK, IU/L	2202 (599, 4195)	2911 (875, 3646)	1830 (576, 4297)	0.65
TIMI 3 Flow, no (%)	80 (93.0)	25 (100.0)	55 (90.2)	0.18
Use of DES, no (%)	67 (77.0)	15 (60.0)	52 (83.9)	0.02
Reperfusion Time, min	270 (199, 489)	248 (200, 410)	282 (198, 506)	0.55
Stent diameter, mm	3.5 (3.0, 3.5)	3.5 (3.0, 3.5)	3.5 (3.0, 3.5)	0.52
Stent length, mm	24 (16, 30)	24 (17, 38)	24 (16, 29)	0.25

Data are expressed as the median (25th, 75th percentiles), or the number (%) of patients.

*BMI* body mass index, *BP* blood pressure, *TIMI* *risk score 2°P* thrombolysis in myocardial infarction risk score for secondary prevention, *HDL-C* high-density lipoprotein cholesterol, *LDL-C* low-density lipoprotein cholesterol, *RLP-C* remnant lipoprotein cholesterol, *Lp(a)* lipoprotein (a), *hsCRP* high sensitive C-reactive protein, *BNP* brain natriuretic peptide, *LVEF* left ventricular ejection fraction, *ACE-I* angiotensin-converting enzyme inhibitor, *ARB* angiotensin II receptor blocker, *PCI* percutaneous coronary intervention, *CPK* creatine phosphokinase, *TIMI* thrombolysis in myocardial infarction, *DES* drug-eluting stent

## Ultrasound measurement of carotid plaque

Ultrasonography of the bilateral common carotid artery (CCA) and internal carotid artery (ICA) was performed during the acute phase, and the intima–media thickness (IMT) was measured as the distance from the leading edge of the lumen-intima interface to the leading edge of the media-adventia interface on a longitudinal image of each carotid artery. Maximum IMT was defined as the greatest IMT of either the left or right CCA and ICA in each patient. After six months, the IMT at the site of maximal IMT was measured to determine whether there was an increase or decrease in IMT. Carotid plaque progression was defined as an increase in IMT from the first to the second test.

## Measurement of epicardial coronary diameter and coronary blood flow response to acetylcholine

Quantitative coronary angiography was performed as previously described [3, 9]. After baseline angiography, incremental doses of ACh (OVISOT, Daiichi Sankyo, Tokyo) (5, 10 and 50 μg/min) were infused directly into the left coronary artery through the Judkins catheter for 2 min with a 5-min interval between successive doses.

To assess the response of the epicardial coronary diameter to ACh, the luminal diameter in a segment located 15–25 mm from the distal edge of the stent in the LAD was measured quantitatively (Cardio 500; Kontron Instruments, Munich, Germany) before and during each infusion [3, 9] Each analyzed segment was referenced to a specific anatomic landmark, including the stent for identification, and analyses at 1–2 weeks and 6 months after AMI were performed in parallel to ensure analysis of the identical portion of the LAD.

Blood flow velocity was measured before and after each infusion using a 0.014-inch wire equipped with a Doppler crystal at its tip (FloWire, Cardiometrics, Mountain View, California) [3, 9]. The wire was advanced through the Judkins catheter, and the wire tip was positioned in a segment of the LAD 5–15 mm from the distal edge of the stent. For flow calculation, the coronary luminal diameter response was measured in a segment of the LAD 5–10 mm distal to the tip of the flow wire before and during each infusion as a separate measurement of the epicardial coronary diameter response to ACh in the LAD, as described above. Coronary blood flow (mL/min) was estimated from the coronary blood flow velocity and luminal diameter using the following formula: 0.5 × average peak velocity (cm/min) × cross-sectional area (cm^2^), as described previously [3, 9]. The responses of coronary artery diameter and blood flow to ACh infusions were expressed as percentage changes from their respective baseline values measured immediately before each infusion. These measurements were performed by two observers (N.T. and Y.T.) who were blinded to the study protocol and patients’ clinical characteristics.

## Determination of the epicardial coronary diameter and coronary blood flow response to ACh in each patient and the cut-off values of impairment of their vasomotor responses

As we previously reported [8, 9], the greatest dilator response from the baseline among the responses to three ACh doses (5, 10 and 50 μg/min) was selected as the epicardial coronary vasomotor response to ACh for each patient. After an additional 15 min, intracoronary sodium nitroprusside (SNP) (10 μg/min) was infused in the same manner as ACh. The epicardial coronary response with the least constriction from the baseline among the three ACh doses was selected for each patient who did not show a dilator response to any ACh dose. Similarly, the greatest increase in the coronary flow response from baseline among the three ACh doses was selected as the coronary flow response to ACh for each patient. The cutoff values of responses to ACh were determined from our previous report, yielding a 4.9% increase from baseline for epicardial coronary dilator response and a 133% increase from baseline for coronary flow response [8, 9].

## Statistical analysis

Data are expressed as median, quartile (first quartile; Q1 and third quartile, Q3), frequency (%), odds ratio (OR), or 95% confidence interval (95% CI). Continuous variables were compared using the Mann–Whitney U and Wilcoxon signed-rank tests, and categorical variables were compared between groups using Pearson’s chi-square analysis or Fisher’s exact test, as appropriate. Correlations between clinical variables were tested using logistic regression analysis, wherein each continuous variable was divided by its standard deviation to calculate the OR. During multivariate analysis, backward stepwise logistic regression was used for variable selection in the study sample. In the multivariate model, we included the following major cardiovascular risk factors in the thrombolysis of myocardial infarction risk score for secondary prevention (TRS 2°P) [14] as covariates: age > 75 years, hypertension, diabetes mellitus, peripheral artery disease, stroke, current smoking, previous heart failure, coronary artery graft bypass, estimated glomerular filtration rate, and carotid plaque progression. Statistical significance was defined as *p* < 0.05. All statistical analyses were performed using SPSS version 26 (IBM Corp., Armonk, NY, USA).

## Results

### Study patients

Total of 87 patients with STEMI who fulfilled the inclusion criteria were included in this study. (Fig. 1). After six months, 25 patients (28.7%) showed carotid plaque progression. The patients’ clinical characteristics are presented in Table 1.

## Comparisons of carotid plaque progression between 1st and 2nd tests

No significant change was noted in the carotid plaque thickness between the first and second tests in the overall patient analysis. The IMT of ICA remained unchanged at 1.0 mm during the second test from the first test. Similarly, the IMT of CCA and maximal IMT changed from 1.2 mm to 1.1 mm and from 1.4 mm to 1.3 mm from the first to the second trial, respectively, but without statistical significance. In the group of patients with plaque progression, a significant increase was noted in the plaque thickness from the first to the second time in both the CCA and ICA, as well as in the maximum IMT. The increase was as follows: 1.0 mm to 1.3 mm, 1.0 mm to 1.3 mm, and 1.3 mm to 1.6 mm, respectively. Conversely, in the group without plaque progression, significant plaque regression was observed from the first to the second session in both CCA, ICA, and maximum IMT, with reductions from 1.2 mm to 1.0 mm, 1.2 mm to 1.0 mm, and 1.5 mm to 1.2 mm, respectively (Supplemental Table 1).

## Comparison of clinical parameters between patients with and without carotid plaque progression

Patients with carotid plaque progression exhibited significantly elevated levels of apolipoprotein C2 and apolipoprotein E at the initial assessment **(**Table 1**)**. Similarly, apolipoprotein C3 and remnant lipoprotein cholesterol levels were significantly higher at the second assessment. In contrast, there was no significant difference in medication use between the initial and second assessments. Among the PCI variables, only the use rate of drug-eluting stent (DES) demonstrated a significant difference.

## Epicardial coronary diameter response and coronary flow response to ACh and carotid plaque progression

In terms of the diameter and flow response, no significant differences were noted between patients with and without carotid plaque progression in the initial test (Table 2).

**Table 2:** Comparisons of Coronary Vasomotor Response between Patients with and without Carotid Plaque Progression

	Total (*n* = 87)	Plaque progression		*p* value
		With (*n* = 25)	Without (*n* = 62)	
Coronary vasomotor response to ACh				
Diameter response at the 1st test	0.5 (− 5.8, 8.2)	-1.1 (-6.4, 4.5)	0.8 (− 5.0, 9.8)	0.29
Diameter response at the 2nd test	9.0 (2.7, 16.8)	3.2 (− 2.2, 18.1)	10.5 (3.8, 16.1)	0.04
Persistent impairment of diameter response	23 (26.4)	11 (44.0)	12 (19.4)	0.02
Flow response at the 1st test	75 (36, 136)	65 (31 (118)	80 (43, 155)	0.46
Flow response at the 2nd test	161 (82, 231)	127 (62, 214)	172 8108, 241)	0.04
Persistent impairment of flow response	28 (32.2)	12 (48.0)	16 (25.8)	0.04
Coronary Vasomotor response to SNP				
Diameter response at the 1st test	2.0 (1.6, 2.2)	2.1 (1.7, 2.3)	1.9 (1.6, 2.2)	0.28
Diameter response at the 2nd test	1.9 (1.6, 2.2)	1.8 (1.6, 2.2)	1.9 (1.7, 2.2)	0.50
Flow response at the 1st test	101 (64, 171)	117 (78, 181)	97 (64, 166)	0.43
Flow response at the 2nd test	165 (88, 235)	199 (90, 253)	157 (87, 234)	0.45

Data are expressed as the median (25th, 75th percentiles).

*ACh* acetylcholine, *SNP* sodium nitroprusside

**Fig. 2:**
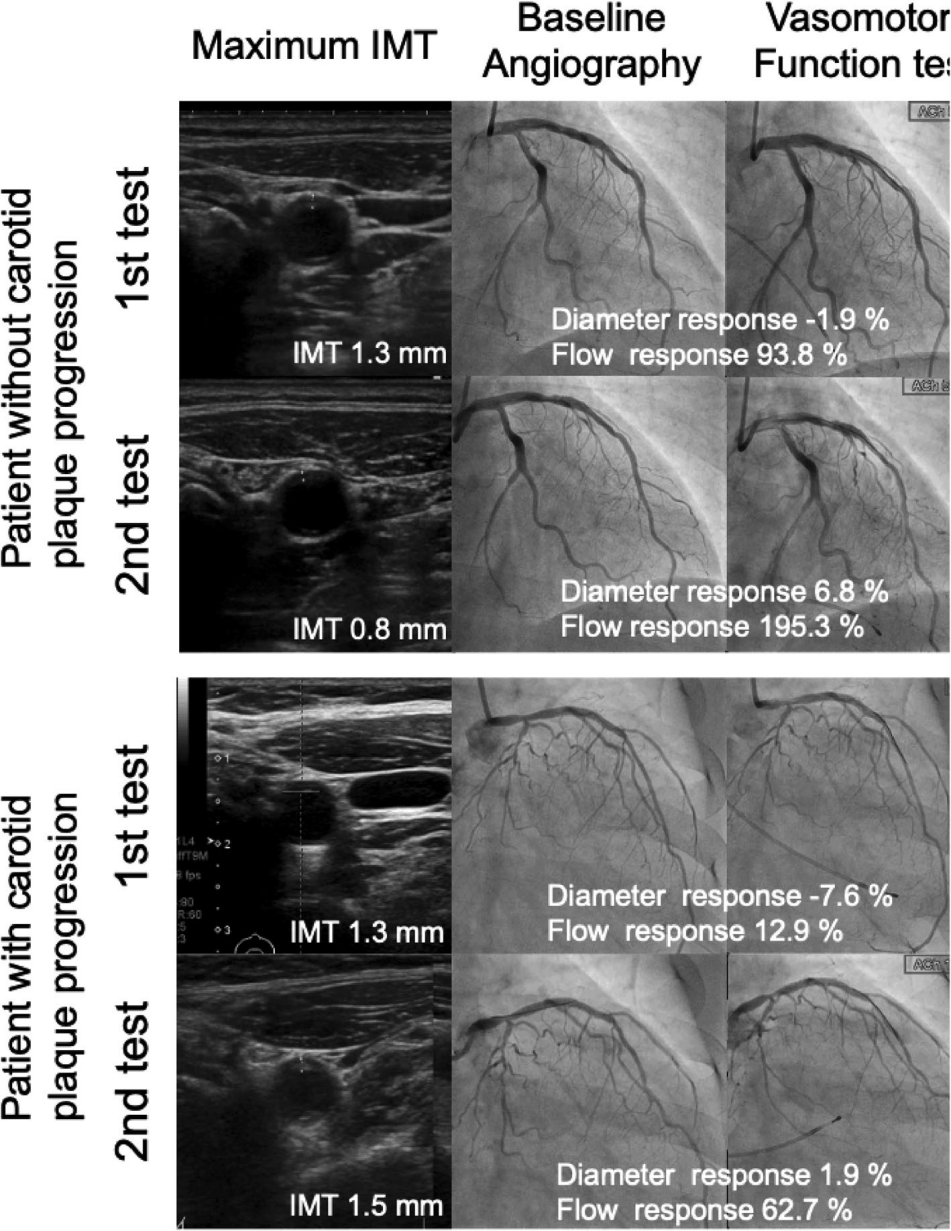
Representative cases of carotid plaque and coronary vasomotor function test at 1st and 2nd examinations

However, in the second test, patients with plaque progression exhibited significantly impaired coronary diameter and flow response compared with those without plaque progression. Moreover, persistent impairment of the diameter and flow response was significantly more prevalent in patients with plaque progression (44.0% vs. 19.4%, *p* = 0.02 for diameter response impairment; 48.0% vs. 25.8%, *p* = 0.04 for flow response impairment, respectively). Representative examples of carotid plaque and coronary vasomotor function tests at the initial and second examinations are shown in Fig. 2. On the other hand, there was no difference in SNP response regardless of the increase or decrease in carotid plaque among initial and second examinations.

## Predicting value of clinical parameters for persistent coronary endothelial dysfunction

In a crude analysis, it was predicted that persistent impairment of coronary diameter response would be associated with TRS 2°P, low-density lipoprotein cholesterol (LDL-C) levels in the second test, brain natriuretic peptide (BNP) levels in the second test, maximum IMT in the second test, diuretic use, and carotid plaque progression. Furthermore, persistent impairment of the coronary flow response was predicted by Apo C2 and Apo C3 in the initial test, BNP levels in the second test, diuretic use in the initial test, smaller stent diameter, and carotid plaque progression (Table 3).

**Table 3:** Crude Logistic regression analysis for predicting persistent coronary endothelial dysfunction

	Diameter response		Flow response	
	OR (95% CI)	*p* value	OR (95% CI)	*p* value
Age, per 10 years	1.11 (0.71—1.75)	0.65	1.15 (0.75—1.77)	0.51
Male gender	0.94 (0.25—3.56)	0.93	0.61 (0.21—1.83)	0.38
Hypertension	1.05 (0.39—2.86)	0.92	2.06 (0.76—5.60)	0.16
Diabetes mellitus	2.09 (0.73—5.99)	0.17	2.42 (0.88—6.68)	0.09
Current smoking	2.13 (0.81—5.64)	0.13	0.78 (0.31—1.92)	0.58
TIMI risk score 2°P	1.91 (1.06—3.43)	0.03	1.59 (0.92—2.72)	0.10
Measurement at 1st test				
Systolic BP	1.03 (0.64—1.66)	0.92	1.22 (0.77—1.91)	0.40
Diastolic BP	0.67 (0.39—1.14)	0.14	0.95 (0.60—1.50)	0.83
HbA1c	1.17 (0.74—1.84)	0.51	1.27 (0.82—1.96)	0.29
Triglyceride	1.17 (0.74—1.85)	0.51	1.18 (0.76—1.83)	0.47
HDL-C	1.08 (0.68—1.74)	0.74	1.28 (0.82—2.00)	0.28
LDL-C	1.07 (0.66—1.73)	0.79	1.03 (0.66—1.62)	0.90
Apo B/A1	1.16 (0.72—1.87)	0.56	0.96 (0.61—1.52)	0.87
Apo C2	1.42 (0.88—2.29)	0.15	1.61 (1.00—2.58)	0.04
Apo C3	1.28 (0.80—2.03)	0.30	1.77 (1.07—2.93)	0.03
Apo E	0.71 (0.41—1.26)	0.24	1.06 (0.68—1.65)	0.81
RLP-C	0.91 (0.56—1.48)	0.70	0.99 (0.63—1.56)	0.97
Lp(a)	1.16 (0.73—1.82)	0.54	1.08 (0.70—1.69)	0.72
hsCRP	1.43 (0.90—2.28)	0.13	1.55 (0.95—2.51)	0.08
BNP	1.47 (0.92—2.34)	0.11	0.67 (0.60—1.56)	0.89
LVEF	1.06 (0.65—1.72)	0.82	0.92 (0.58—1.45)	0.72
Max IMT	0.13 (0.77—1.95)	0.39	0.96 (0.61—1.51)	0.85
Measurement at 2nd test				
Systolic BP	0.72 (0.43—1.20)	0.20	0.75 (0.46—1.20)	0.23
Diastolic BP	0.89 (0.55—1.44)	0.63	0.90 (0.57—1.41)	0.63
HbA1c	1.34 (0.85—2.10)	0.21	1.36 (0.88—2.12)	0.17
Triglyceride	1.19 (0.76—1.85)	0.45	1.03 (0.66—1.61)	0.90
HDL-C	1.12 (0.69—1.84)	0.65	1.04 (0.65—1.66)	0.88
LDL-C	1.90 (1.08—3.33)	0.03	1.29 (0.79—2.08)	0.31
Apo B/A1	1.11 (0.72—1.71)	0.65	0.86 (0.47—1.58)	0.62
Apo C2	1.54 (0.95—2.48)	0.08	1.28 (0.82—1.99)	0.28
Apo C3	1.33 (0.85—2.08)	0.21	1.20 (0.78—1.85)	0.41
Apo E	1.10 (0.70—1.73)	0.67	0.96 (0.60—1.52)	0.85
RLP-C	1.06 (0.68—1.65)	0.81	0.99 (0.63—1.57)	0.99
Lp(a)	1.29 (0.82—2.03)	0.26	1.39 (0.89—2.16)	0.15
hsCRP	1.40 (0.86—2.29)	0.18	1.26 (0.77—2.05)	0.35
BNP	1.88 (1.13—3.15)	0.02	1.99 (1.14—3.47)	0.02
LVEF	1.03 (0.64—1.66)	0.91	0.95 (0.60—1.49)	0.82
Max IMT	1.73 (1.08—2.78)	0.02	1.20 (0.77—1.87)	0.42
Carotid Plaque progression	3.27 (1.19—8.99)	0.02	2.65 (1.01—7.00)	0.04
Medications at 1st test				
Beta-blocker	1.05 (0.40—2.76)	0.91	1.26 (0.51—3.13)	0.61
ACE-I/ARB	0.79 (0.29—2.18)	0.65	1.19 (0.44—3.18)	0.73
Ca blocker	1.37 (0.49—3.81)	0.55	0.99 (0.39—2.54)	0.99
Statin	0.89 (0.16—4.94)	0.89	3.06 (0.35—26.69)	0.31
Diuretics	5.65 (1.23—25.95)	0.03	7.77 (1.46—41.46)	0.02
Insulin	2.86 (0.17—47.75)	0.46	2.15 (0.13—35.65)	0.59
Medications at 2nd test				
Beta-blocker	1.50 (0.57—3.89)	0.41	1.36 (0.55—3.36)	0.51
ACE-I/ARB	0.57 (0.20—1.62)	0.29	0.85 (0.31—2.34)	0.76
Ca blocker	1.04 (0.37—2.92)	0.94	1.19 (0.44—3.18)	0.73
Statin	0.34 (0.05—2.56)	0.29	N/A	-
Diuretics	4.23 (1.25—14.37)	0.02	2.94 (0.89—9.79)	0.08
Insulin	2.86 (0.17—47.75)	0.46	2.15 (0.13—35.65)	0.59
PCI variables				
Peak CPK	1.04 (1.00—1.09)	0.07	1.00 (0.96—1.04)	0.99
TIMI 3 Flow	0.71 (0.12—4.18)	0.71	2.55 (0.28—22.91)	0.40
Use of DES	0.58 (0.20—1.71)	0.33	0.85 (0.30—2.43)	0.76
Reperfusion time, per 30 min	1.02 (0.95—1.09)	0.60	1.02 (0.96—1.10)	0.49
Stent diameter, per 0.1 mm	0.97 (0.85—1.09)	0.58	0.87 (0.77—0.98)	0.03
Stent length, per 1 mm	1.01 (0.96—1.05)	0.82	1.03 (0.98—1.07)	0.23

Data expressed as odd ratio (OR) and 95% confidential interval (95%CI). Abbreviation: IMT, intima media thickness, other abbreviations are the same as in Table 1

In a multivariable-adjusted analysis, carotid plaque progression was identified as an independent predictor of persistent coronary endothelial dysfunction, both in terms of coronary diameter response (OR 3.22, 95% CI 1.13—9.15, *p* = 0.03) and coronary flow response (OR 2.65, 95% CI 1.01—7.00, *p* = 0.04). These findings are presented in Table 4.

**Table 4:** Adjusted Logistic regression analysis for predicting persistent coronary endothelial dysfunction

	Diameter response		Flow response	
	OR (95% CI)	*p* value	OR (95% CI)	*p* value
TIMI risk score 2°P	1.89 (1.03—3.47)	0.04	Not selected	–
Carotid plaque progression	3.22 (1.13—9.15)	0.03	2.65 (1.01—7.00)	0.04

Data expressed as odd ratio and 95% confidential interval, TIMI risk score 2°P include following covariates: age over 75 yrs, hypertension, diabetes mellitus, peripheral artery disease, stroke, current smoking, previous heart failure, coronary artery graft bypass, estimated glomerular filtration rate

## Discussion

This clinical study showed that carotid plaque progression is associated with persistent endothelial vasomotor dysfunction of IRA in STEMI survivors. The vascular endothelium produces nitric oxide (NO) as an endothelium-dependent vasorelaxant factor [15] that exerts myocardial protective effects via anti-inflammatory, anti-apoptotic, and anti-fibrotic effects [16–18]. Myocardial infarction itself is known to provoke coronary artery endothelial dysfunction [19], and it has been reported that reperfusion injury caused by revascularization may further accelerate endothelial dysfunctionb [2, 5, 20, 21]. Endothelial dysfunction results in decreased NO production and function [22, 23], which has been reported to be associated with future cardiovascular events due to reduced cardio-protection [24, 25]. In this study, the frequency of DES implantation was found to be significantly lower in patients with carotid plaque progression. Given these findings, the influence of systemic exposure to the drug resulting from DES implantation should not be given much consideration, as previous research has indicated that the drug is undetectable in the blood during the long-term phase [26, 27]. The enrollment period was lengthy, and the percentage of patients with DES implantation was merely 77%. This figure is considered low from the current clinical perspective, and it is possible that various unmeasured factors have influenced the outcome.

In addition, coronary endothelial function can be indirectly assessed using FMD of the brachial artery [28, 29], and an association has been reported between endothelial function examination using FMD and carotid artery plaque [30]. On the other hand, the evaluation of carotid plaques by ultrasound is simpler than FMD and is a widely recognized and performed examination. Carotid and coronary plaques are closely related and inseparable as part of the same vascular system [31].

We have shown that coronary endothelial dysfunction after myocardial infarction, which does not improve and persists into the chronic phase, is an independent or of increased coronary plaques and cardiovascular events [8, 9]. In our previous studies, endothelial function was assessed invasively; however, it is easier to estimate endothelial function in a noninvasive manner for routine clinical use.

As noted above, FMD testing is an indirect assessment of vascular endothelial function; however, a simpler test is desirable because of the proficiency of the measurement machine and technique. In this study, we showed that noninvasive evaluation of carotid plaques is a strong and independent predictor of persistent coronary endothelial dysfunction in the chronic phase. Furthermore, it has been recently reported that an improvement in vascular endothelial function over time after AMI is significantly and independently associated with an improvement in quality of life [32]. Therefore, it is significant that endothelial dysfunction can be more easily and indirectly assessed.

Carotid plaque progression had a significant association with persistent coronary endothelial dysfunction in the multivariate model adjusted for TRS2°P, including various conventional risk factors. Therefore, even with optimal medical therapy, patients with carotid plaque progression in the chronic phase may be at high risk of future cardiovascular events, and there is a need to consider more aggressive medical interventions for these patients.

## Study limitation

Although this was a prospective study, the possibility of selection bias cannot be ruled out because it was a non-randomized trial. Additionally, the possibility of unknown confounding factors that could not be measured cannot be ruled out. Therefore, this study used a multivariate model to eliminate bias as much as possible. Furthermore, the patient inclusion period in this study was long, ranging from 2007 to 2017, and the effect of advances in catheter devices and drug therapy updates during this period cannot be excluded. Therefore, a prospective, larger, and shorter study is required. Although this research constitutes a sub-analysis, it has demonstrated a significant, independent relationship between the progression of carotid plaque and persistent endothelial dysfunction. However, additional prospective studies are necessary to determine whether this relationship is causal or consequential.

## Conclusion

In conclusion, carotid plaque progression had a significant association with persistent endothelial vasomotor dysfunction in the IRA of STEMI survivors. More intensive care might be needed in patients with carotid plaque progression undergoing OMT to improve coronary endothelial function.

## Supplementary Information

Below is the link to the electronic supplementary material.

Supplementary file1 (DOCX 15 KB)
