# Supplementary material for: Association between carotid plaque progression and persistent endothelial dysfunction in an infarct-related coronary artery in STEMI survivors
Source: Heart Vessels. 2024 Jul 27;40(1):36–46. doi: 10.1007/s00380-024-02444-z (PMC11717882; doi:10.1007/s00380-024-02444-z)
Supplement: Supplementary file 1 — Supplementary file1 (DOCX 15 KB) [file 380_2024_2444_MOESM1_ESM.docx]

| **Supplemental Table 1. Comparisons of Carotid Plaque between 1^st^ and 2^nd^ test** | | | | |
| --- | --- | --- | --- | --- |
|  |  | 1^st^ test | 2^nd^ test | *p* value |
| *Over all patients* | |  |  |  |
|  | Internal Carotid Plaque, mm | 1.0 (0.8, 1.8) | 1.0 (0.7, 1.7) | 0.13 |
|  | Common Carotid Plaque, mm | 1.2 (0.8, 1.8) | 1.1 (0.8, 1.7) | 0.66 |
|  | Maximum Carotid Plaque, mm | 1.4 (1.0, 2.2) | 1.3 (0.9, 2.0) | 0.07 |
|  | |  |  |  |
| *With Plaque progression* | |  |  |  |
|  | Internal Carotid Plaque, mm | 1.0 (0.7, 1.8) | 1.3 (0.8, 1.8) | <0.001 |
|  | Common Carotid Plaque, mm | 1.0 (0.8, 1.8) | 1.3 (0.9, 2.3) | <0.001 |
|  | Maximum Carotid Plaque, mm | 1.3 (0.9, 2.3) | 1.6 (1.3, 2.7) | <0.001 |
|  | |  |  |  |
| *Without Plaque progression* | |  |  |  |
|  | Internal Carotid Plaque, mm | 1.2 (0.8, 1.8) | 1.0 (0.7, 1.6) | <0.001 |
|  | Common Carotid Plaque, mm | 1.2 (0.9, 1.8) | 1.0 (0.8, 1.5) | 0.001 |
|  | Maximum Carotid Plaque, mm | 1.5 (1.0, 2.0) | 1.2 (0.8, 1.8) | <0.001 |

Data are expressed as the median (25th, 75th percentiles).
